# Transportability of tertiary qualifications and CPD: A continuing challenge for the global health workforce

**DOI:** 10.1186/1472-6920-12-51

**Published:** 2012-07-09

**Authors:** Deborah C Saltman, Michael R Kidd, Debra Jackson, Michelle Cleary

**Affiliations:** 1 Faculty of Nursing, Midwifery and Health, University of Technology Sydney (UTS), Sydney, Australia; 2 Discipline of General Practice, Central Clinical School, Sydney Medical School, The University of Sydney, Sydney, Australia; 3 Faculty of Health Sciences, Flinders University, Adelaide, Australia; 4 School of Nursing and Midwifery, University of Western Sydney, Sydney, Australia; 5 Alice Lee Centre for Nursing Studies, Yong Loo Lin School of Medicine, Singapore, Singapore

**Keywords:** Health workforce, Continuing professional development, Health workforce education discursive paper

## Abstract

**Background:**

In workforces that are traditionally mobile and have long lead times for new supply, such as health, effective global indicators of tertiary education are increasingly essential. Difficulties with transportability of qualifications and cross-accreditation are now recognised as key barriers to meeting the rapidly shifting international demands for health care providers. The plethora of mixed education and service arrangements poses challenges for employers and regulators, let alone patients; in determining equivalence of training and competency between individuals, institutions and geographical locations.

**Discussion:**

This paper outlines the shortfall of the current indicators in assisting the process of global certification and competency recognition in the health care workforce. Using Organisation for Economic Cooperation and Development (OECD) data we highlight how International standardisation in the tertiary education sector is problematic for the global health workforce. Through a series of case studies, we then describe a model which enables institutions to compare themselves internally and with others internationally using bespoke or prioritised parameters rather than standards.

**Summary:**

The mobility of the global health workforce means that transportability of qualifications is an increasing area of concern. Valid qualifications based on workplace learning and assessment requires at least some variables to be benchmarked in order to judge performance.

## Background

In workforces that are traditionally mobile and have long lead times for new supply, such as health, global indicators of tertiary education are increasingly important. Issues associated with cross-accreditation and transportability of qualifications are now recognised as key barriers in facilitating the efficacious movement of health care providers and meeting rapidly shifting international demands. Traditionally, the arbiters of equivalence and cross-accreditation have been regulatory authorities. However, as the education of health workers becomes based more in universities and other tertiary institutions, it is the historical competence and sophistication of universities that ensures academic accountability. In relation to some courses, Universities are relied upon to certify that their programmes translate easily between regulators, and ensure competency between services both locally and internationally. Paradoxically this accountability now extends beyond customary university precincts, as the emphasis in health professional education increasingly becomes focussed on learner-centred activities which are delivered in off-campus, vocational environments [1].

The shift from formal course development and student completion to competency and performance on-the-job is even more pronounced in the postgraduate arena. For example in the English-speaking countries, the task of delivering preparatory, specialist and continuing education programmes has been devolved to three groups: academic institutions such as universities, professional organisations such as colleges and academies, and/or service providers such as health authorities. Each of these groups operates within a different sector, and there may be little synergy or collaborative effort between educational providers, clinical settings and regulatory authorities. Furthermore, educational providers – even those within the same group - can have vastly differing ideologies and educational practices, which shape how curricula is formed, how learning and teaching is conceived and delivered, and thus exert a powerful influence on graduate outcomes [2]. While within countries/states there are often arbiters/regulators with an agenda to ensure local consistency between ‘like’ courses; to date there have been only sporadic attempts by relevant accrediting bodies to map competencies across regions or countries.

One of the major difficulties is that many different mixed models are in existence. The plethora of mixed education and service arrangements poses challenges for employers and regulators; let alone patients in determining equivalence of training and competency between individuals, institutions and geographical locations. This paper outlines the shortfall of the current indicators in assisting the process of global certification and competency recognition in health care. Using OECD data we highlight how International standardisation in the tertiary education sector is problematic. Through a series of case studies, we then describe a model which enables institutions to compare themselves internally and with others internationally using bespoke or prioritised parameters rather than standards.

### The paradoxes of current benchmarking and standards in facilitating transportability

Certainly, in an ideal world, a continuous process for systematically evaluating the products, services and work of educational organisations would provide a gold standard which could then be incorporated into continuous quality improvement equally in all sectors of endeavour [3]. To date tertiary education’s capacity to confer an employment advantage has been one such gold standard. In reality the picture is far from clear cut. Our first case study highlights the use of unemployment advantage as an outcome measure for tertiary education.

### Case study 1A: unemployment advantage: an OECD artefact?

In the decade 1995–2004 the Organisation for Economic Cooperation and Development (OECD) collected data from member nations about the impact of tertiary education. The data gathered was essentially a measure of undergraduate activity and its direct impact on employment and social markers. The outcome of the research was expected to show a positive relationship. However, in OECD 2002, the Main Science and Technology Indicators and Education Indicators highlighted the unpredictability of this association with several countries showing negative or unclear relationships. In 12 OECD countries the mean unemployment rates were significantly decreased (ANOVA with sphericity assumed F = 14.98; P < 0.0005) from 1991 to 2002 in the following 12 countries: Denmark, Hungary, United Kingdom, Australia, Portugal, Norway, Turkey, Ireland, Mexico, Spain, Netherlands and New Zealand. In three countries, Japan, Germany and Czech Republic, the mean unemployment rates were significantly increased (ANOVA with sphericity assumed P < 0.0005) from 1991 to 2002. In the remaining 15 countries the pattern has been mixed. In reality, there are too many externalities that corrupt any sense of causality between education attainment and unemployment rates. See Table 1 for trends in unemployment advantage from 1991–2002.

**Table 1:** Trends in unemployment advantage 1991-2002*

**Countries**	**Trends in Unemployment Advantage (1991–2002)**							**Mean + SD**	*r*	*P*
	**1991**	**1995**	**1998**	**1999**	**2000**	**2001**	**2002**			
Sweden	1	3.7	2.8	2.1	1.9	1.6	1.2	2.04 + 0.94	−0.120	*0.798*
France	2.2	1.8	2.2	2.3	2	1.5	1	1.85 + 0.46	−0.557	*0.194*
Canada	1.6	1.4	1.9	1.5	1.2	1	1.1	1.38 + 0.31	−0.570	*0.184*
Belgium	2.7	3	2.5	1.8	1.9	1.7	.	2.17 + 0.55	−0.055	*0.907*
Finland	3	5.9	3.6	3.7	3.2	3.1	3.2	3.67 + 1.01	−0.278	*0.545*
United States	2.6	1.6	1.7	1.2	1.3	1.3	1.9	1.65 + 0.48	−0.690	*0.087*
Italy	1.1	−0.4	0.3	0.1	0.5	0.6	0.4	0.37 + 0.46	−0.189	*0.685*
Luxembourg	.	.	.	0	0.3	−0.3	−0.6	−0.15 + 0.38	−0.800	*0.200*
Denmark	3.4	4.2	0.9	0.7	0.9	−0.2	−0.4	1.35 + 1.76	−0.892	*0.007*
Hungary	.	.	3.3	3.3	3	2.4	2.1	2.82 + 0.54	−0.957	*0.010*
UnitedKingdom	2.5	2.8	1.8	1.7	1.9	1.4	1.2	1.90 + 0.57	−0.860	*0.011*
Australia	1.9	1.5	1.8	1.3	0.6	1.2	0.7	1.28 + 0.50	−0.772	*0.042*
Portugal	2.3	2.3	1.7	1	0.5	0.3	0.1	1.17 + 0.93	−0.920	*0.003*
Norway	1.9	1.2	0.6	0.9	0.5	0.8	0.5	0.91 + 0.50	−0.920	*0.003*
Turkey	2.4	2.2	0.6	1.5	0.6	1.2	−0.2	1.18 + 0.93	−0.836	*0.019*
Ireland	1.5	1.8	0.7	1.3	0.5	0.7	0.6	1.01 + 0.51	−0.775	*0.041*
Mexico	0.8	0.5	0.3	0.2	0.4	0.	2.	0.40 + 0.22	−0.919	*0.010*
Spain	2	2	0.6	0.7	0.6	0.6	0.7	1.02 + 0.66	−0.883	*0.008*
Netherlands	2.3	0.2	.	0.4	0.1	0.3	−0.1	0.53 + 0.88	−0.843	*0.035*
New Zealand	1.7	0.1	0.2	0.4	−0.1	0	−0.1	0.31 + 0.63	−0.848	*0.016*
Greece	−0.8	2.3	1.4	1.7	1.4	1.4	.	1.23 + 1.05	0.627	*0.182*
Austria	1	0.5	1.1	0.9	0.9	1	1.1	0.92 + 0.20	0.338	*0.458*
Korea	−0.8	−0.5	0.9	0.8	0.1	−0.2	−0.4	−0.01 + 0.65	0.394	*0.382*
Switzerland	0	0.5	−0.3	0.3	0.5	0.5	−0.1	0.20 + 0.33	0.118	*0.801*
Iceland	.	0.4	0.3	0.6	0.8	0.9	.	0.60 + 0.25	0.809	*0.097*
Poland	6.3	4.9	5.6	6.9	7.8	8.2	.	6.61 + 1.27	0.624	*0.185*
Slovak Republic	.	5.6	4.2	6.1	7.7	8.3	8.5	6.73 + 1.70	0.776	*0.070*
Japan	.	0.4	0.7	0.7	1.1	0.9	.	0.76 + 0.26	0.883	*0.047*
Germany	0.8	1.8	3	2.4	2.5	2.6	3	2.30 + 0.77	0.908	*0.005*
Czech Republic	.	1.2	2	2.9	3.3	3.2	2.9	2.58 + 0.81	0.887	*0.018*
**Mean + SD**	1.69 + 0.23	1.81 + 0.36	1.37 + 0.23	1.30 + 0.19	1.05 + 0.19	1.00 + 0.19	0.81 + 0.23			
**95 % CI**	[1.19 - 2.18]	[1.05 - 2.57]	[0.87 - 1.87]	[0.88 - 1.72]	[0.64 - 1.47]	[0.59 - 1.40]	[0.32 - 1.30]			

^*^Table adapted from Hase [4].

In 2005, a number of more granular indicators were introduced. These new markers included measures of: input, process and output; diversity and inclusivity; engagement and learning community; and assessment [5]. This increased granularity and flexibility in the indictors, led to the local development of a basket of quality indicators from which individual universities could select elements most appropriate to their individual circumstances [6]. To date and to the best of our knowledge, these indicators have not been used for comparisons in the global arena.

### Case study 1B: unemployment advantage as an outcome measure in medicine and nursing

Within disciplines where basic training is highly regulated, for example medicine, nursing and pharmacy, workforce factors and demand influence employment outcomes. Table 2 shows the level of employment at 6 months for graduates in medicine, nursing, pharmacy and biosciences from UK Universities [7,8]. In the case of the very specific example of medicine, where there are relatively few courses and the financial advantage of the degree is high; demand outstrips supply and overall employment is high. Conversely, where a course is not regulated and many institutions produce graduates with varying competencies, such as Biosciences, employment is much lower. In the case of nursing, where there are a plethora of institutions providing a regulated course with reasonable financial prospects, the impact of a particular institution begins to emerge. The wide range of employment percentages between universities suggests that some discrimination conferring work advantage may occur in this group of graduates.

**Table 2:** Employment at 6 months for graduates in medicine, nursing, pharmacy and biosciences from UK Universities*

Degree	% Students with Job after 6 months	Range %	Number of Universities with Course in UK
Medicine	100	98-100	30
Nursing and paramedical	96	62-100	74
Pharmacy	89	72-100	28
Biosciences	65	41-83	91

^*^Data derived from The Guardian (2006) http://www.guardian.co.uk/education/2006/may/02/universityguide2?INTCMP=SRCH.

### Case study 2: postgraduate qualifications: benchmarking high achievers

Whilst undergraduate courses are generally regulated by tertiary education authorities and supervisory bodies, the same cannot be said for postgraduate and continuing professional development (CPD). There is much variability in inter-country and inter-professional education. Whilst we have focussed on competent completion (as inferred by the attainment of a University degree) which may (or may not) result in an increased likelihood of employment, there must also be the ability to recognise and benchmark high achievement. Differentiating levels of achievement above the norm distinguishes competency from excellence. For example in postgraduate medical education, the baseline entry level for a programme would be recognition of the educational worthiness of the course - usually achieved within organisations by internal and external curricular review. Standardisation would occur where completion of the course confers a level of training acceptable to an external body e.g. a College or Academy. However, the requirement of a certain qualification to confer professional credentialing by a College or Academy does not necessarily imply that the qualification is subject to any real scrutiny by the conferring College. The provision of an exemplary course may only be assessed by a basket of less defined outcomes, such as for example, post-course employment options and mandatory continuing professional development.

**Figure 1:**
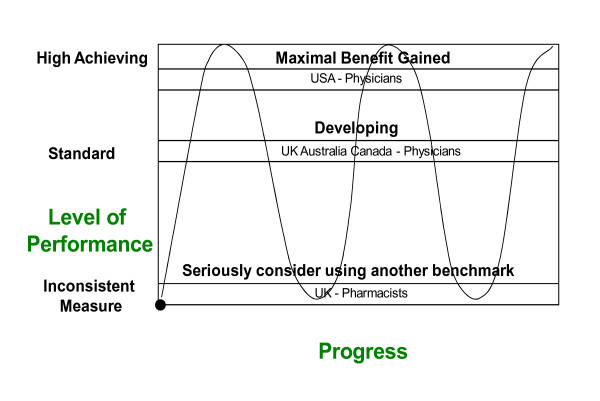
CPD as an intercountry measure.

In CPD the delineation between countries and professional groups is emerging. For example, in the United States of America, the provision of CPD for physicians is regulated by a national agency: The Accreditation Council for Continuing Medical Education (ACCME). In 2009, there were over 700 accredited provider organisations with a gross annual income of over US$2 billion providing courses for over 10 million participants [9]. Whereas in the United Kingdom, where recertification is under development, incomplete data from one commercial site reveals as at August 2010: 208 medical courses (14 accredited), 361 nursing courses (6 accredited), 91 pharmacy courses (3 accredited) and 28 biosciences courses (8 accredited)[10]. When considering CPD, countries can be classified into three groups: high achieving; benefit at standard and maintained; developing or inconsistent benefit. For the latter group CPD may not be a suitable parameter for comparison (see Figure 1).

Using a wave analogy, high achieving countries or professional groups may be compared. The level of achievement in this process may be measured according to whether maximal benefit has been gained independent of International standardised data (for example between ACCME accredited providers in the USA) or where the achievement is stabilised at a developing level (as in the Canada, UK and Australia, as per data presented previously in Table 1). Inconsistent achievement over time in this parameter (e.g. Pharmacy) would exclude this item in a transportability of qualifications analysis. In this way it is possible to effectively measure across domains and cultures in a flexible and comprehensible way. This becomes very important when comparisons are made internationally, for example between English-speaking specialty training programmes (USA, UK and the “Commonwealth” countries) or across regional programmes within spheres of influence, where the commonality is geographical or political, rather than cultural, such as the European Union (EU). For example in the EU, academic and professional medical degrees in recognised defined areas are fully cross-border transferable (Directive 2005/36/EC as of 20 October 2007). Similarly, from a nursing perspective, nurses who may not previously have been able to obtain international registration are now able to do so because of EU agreements.

### Case study 3: multiple measures

**Figure 2:**
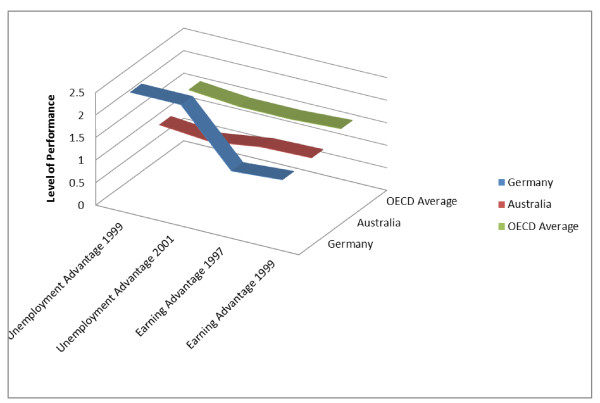
Multiple measures^*^. *Sources for Figure 2 drawn from Hase [4].

There are inherent risks in focussing across all areas of comparison in a systematic way. For example, if an institution that performs above average in trends in unemployment advantage but below average in terms of earning advantage (Germany) were compared with an institution where the converse is true, that is, poor unemployment advantage; high earning advantage (Australia), comparisons may be made across domains where achievements either need no improvement or the degree of comparative improvement is unrealistic. Also, there is a risk that over time whilst there may be an improvement in the institutions in their weaker domains, there may be an inappropriate regression for both institutions in the areas of better than average performance. The situation can also be confounded when an institution achieving high results across a number of domains is compared with others with lower results. We highlight this phenomenon in Figure 2 where we figuratively show these two countries in terms of their performance in these two indicators indicators (as per data presented previously in Table 1 drawn from Hase [4]).

The gap between a relatively poor performing indicator in one institution against another institution with a relatively high value for that indicator may be too wide and result in inhibiting change rather than facilitating it. If the better performing institution is too robust, it may be unlikely that improvement will occur in the poorer performing institution. The weaker performer may lose motivation to change if the perceived gap is too large. Thus, not only must the comparator component be appropriately measured across institutions but also the stronger partner must be committed to assisting the weaker partner to improve its performance, where improvement is within the realms of possibility. This is essentially a process of mentor benchmarking and is useful to raise institutions to the level of a recognised standard.

### Steps to effective transportability of qualifications by individuals

There will always be a dissonance between institutional objectives and their mission statements, and what can be effectively benchmarked against other institutions or graduates. For the graduates and postgraduates, who will no doubt be moving from workplace to workplace in their professional lives, transportability of qualifications will be essential to ensure that their skills are recognised. It has been argued that the outcomes-based method of determining occupational competence moves the focus from what skills the individual ought to hold, to how he or she is expected to function in the workplace [11]. This de-linking of assessments and standards permits for a range of alternate learning practices including experiential learning to be professionally recognised and accredited in relation to a qualification. The importance of an individual student portfolio in fulfilling this role is gaining currency and cannot be underplayed [12].

### Case study 4: mapping academic currency

A greater focus on competency will mean that ‘academic currency’, a term used to describe courses that transfer credit to degrees or confer work advantage will have a greater influence on course content. Some observers now argue that certification is viewed more favourably than a degree [13]. Higher qualifications such as diplomas and masters degrees are not as important to employers as are actual knowledge, competent performance, and appropriate skills. This is increasingly apparent in the UK where the regulators of vocational training in medicine have decreed that all hospital-based clinicians who wish to teach medical students and trainees must have educational certification.

**Figure 3:**
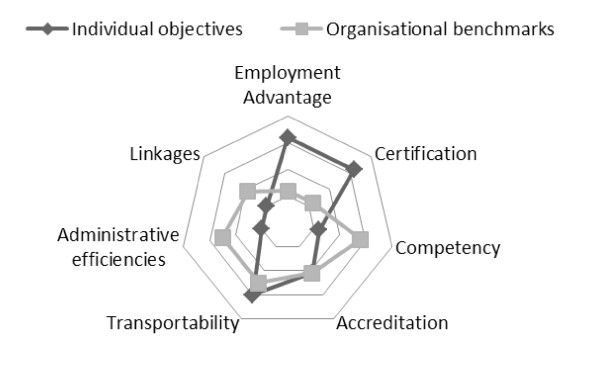
Individual objectives and organisational benchmarks.

Figure 3 demonstrates how organisations may value differing benchmarks to the individuals enrolled in their programmes. These features can be mapped against other individuals and organisations as priorities shift. The outside shell features the characteristics of organisational benchmarking. The light grey dots show organisational concerns and the value providers place on the various characteristics, while the black dots represent the value placed on these same characteristics by the student or learner.

### Summary

The challenge for health professional learners and their current and prospective employers is to establish cross-institutional referencing for their work-based learning. Institutional accreditation and individual certification may not be enough, given the complexity and considerable variance that occurs in areas that traditionally offer academic credibility such as examination and simulated skill-testing systems. Legitimate qualifications grounded in workplace based learning and assessment requires at least some variables to be benchmarked in order to judge performance. These developments have led more tertiary institutions to create vocational training partnerships to share expertise and to produce and deliver courses.

## Competing interests

The author(s) declare that they have no competing interests. The authors alone are responsible for the content and writing of the paper.

## Authors’ contributions

DCS conceived the paper, collected the data and DCS, MK, DJ and MC participated in critical review and preparation of the manuscript. All authors read and approved the final manuscript.

## Disclosures

None for any author

## Pre-publication history

The pre-publication history for this paper can be accessed here:

http://www.biomedcentral.com/1472-6920/12/51/prepub

## References

[B1] Cleary M The views of mental health nurses on continuing professional development J Clin Nurs 2011 20 23–24 3561 3566 21722221 10.1111/j.1365-2702.2011.03745.x

[B2] Davenport N Spath M Blauvelt M A step-by-step approach to curriculum review Nurse Educ 2009 34 4 181 185 10.1097/NNE.0b013e3181aaba80 19574860

[B3] Spendolini M The benchmarking book 1992 Amacom, New York

[B4] Hase H OECD Thematic Review of Tertiary Education Comparative Indicators on Tertiary Education; 1st Workshop of Participating Countries. OECD Directorate for Education: Education and Training Policy Division 2005 www.oecd.org/dataoecd/56/63/35940816.ppt

[B5] OECD Education at a glance: OECD indicators 2005 2005 520 http://www.oecd.org/document/34/0,3746,en_2649_39263238_35289570_1_1_1_1,00.html

[B6] Chalmers D A review of Australian and international quality systems and indicators of learning and teaching 2007 Australian Learning and Teaching Council (ALTC),

[B7] The Guardian The Guardian university guide 2010 http://www.guardian.co.uk/education/

[B8] Guardian The data - where it comes from and what it means. In The Guardian May 2nd 2006, London http://www.guardian.co.uk/education/2006/may/02/universityguide2?INTCMP=SRCH

[B9] ACCME ACCME Annual Report Data 2009 2009 http://www.accme.org/dir_docs/doc_upload/f2e89864-b4c1-428f-8ebe-1ba197a31928_uploaddocument.pdf

[B10] CPDWebsite 2010 http://www.findcpd.com/search/

[B11] Leung D Kember D The influence of teaching approach and teacher-student interaction on the development of graduate capabilities Structural Equation Modeling 2006 13 164 186

[B12] Saltman D Tavabie A Kidd M The use of reflective and reasoned portfolios by doctors J Eval Clin Pract 2012 18 1 182 185 10.1111/j.1365-2753.2010.01514.x 20666885

[B13] Gallagher R The next 20 years: How is online distance learning likely to evolve? In UCEA 88th Annual Conference 2003, Chicago, Illinois

